# Investigation of *Pseudomonas aeruginosa* strain PcyII-10 variants resisting infection by N4-like phage Ab09 in search for genes involved in phage adsorption

**DOI:** 10.1371/journal.pone.0215456

**Published:** 2019-04-16

**Authors:** Libera Latino, Cédric Midoux, Gilles Vergnaud, Christine Pourcel

**Affiliations:** Institute for Integrative Biology of the Cell (I2BC), CEA, CNRS, Univ. Paris-Sud, Université Paris-Saclay, Gif-sur-Yvette cedex, France; Emory University School of Medicine, UNITED STATES

## Abstract

Bacteria and their bacteriophages coexist and coevolve for the benefit of both in a mutualistic association. Multiple mechanisms are used by bacteria to resist phages in a trade-off between survival and maintenance of fitness. *In vitro* studies allow inquiring into the fate of virus and host in different conditions aimed at mimicking natural environment. We analyse here the mutations emerging in a clinical *Pseudomonas aeruginosa* strain in response to infection by Ab09, a N4-like lytic podovirus and describe a variety of chromosomal deletions and mutations conferring resistance. Some deletions result from illegitimate recombination taking place during long-term maintenance of the phage genome. Phage variants with mutations in a tail fiber gene are selected during pseudolysogeny with the capacity to infect resistant cells and produce large plaques. These results highlight the complex host/phage association and suggest that phage Ab09 promotes bacterial chromosome rearrangements. Finally this study points to the possible role of two bacterial genes in Ab09 phage adhesion to the cell, *rpsB* encoding protein S2 of the 30S ribosomal subunit and *ORF1587* encoding a Wzy-like membrane protein involved in LPS biosynthesis.

## Introduction

Bacteriophages play an important role in shaping the bacterial genome by exerting a selective pressure [1]. Bacterial resistance to bacteriophage is principally mediated by modification of the phage receptors although other mechanisms can play a role by blocking the viral entry and multiplication [2]. Recently a large number of new antiphage defense systems present in multicomponent defense islands (among which restriction-modification and CRISPR-Cas systems) have been described [3,4]. In return phage counteracts the defense mechanisms by developing different strategies [5]. Long-term phage/bacteria arm-race has been extensively investigated in the laboratory, mostly using coevolution in chemostats, and a few studies have observed coevolution in natural environment [6,7]. In the environment, many factors influence the fate of bacteria and phage variants by affecting the fitness as observed with *Pseudomonas fluorescens* for example [8]. In *P*. *aeruginosa* the filamentous Pf4 prophage is essential for several stages of the biofilm life-cycle and contributes to the bacterial virulence [9]. Adaptation of a bacterial strain to stress or to a particular niche may be accompanied by important rearrangements of the genome. Reduction of the genomic repertoire is the result of large deletions caused by homologous and illegitimate recombination during permanent association of pathogenic bacteria with their host [10]. Recombination between IS elements or prophages have been shown to be the most frequent mechanism for inversions and deletions [11,12,13]. Another actor in phage/bacteria evolution is the RecA-dependent spontaneous prophage induction (SPI) that influence bacterial fitness [14]. This may be linked to SOS response but also results from other mechanisms.

Whereas the effect of prophage on their host has been widely described, little is known about the effect of lytic phages on the natural rate of evolution, principally due to lack of experimental systems to observe such interactions. *In vitro* investigations have shown that bacterial resistance to phages could modify their virulence [15]. We previously set-up an assay to analyze the mechanisms of resistance developed by the reference *P*. *aeruginosa* strain PAO1 following infection by lytic phages [16]. In this assay which allowed the analysis of a micropopulation of resistant bacteria, we found that pseudolysogeny played an important role in allowing the maintenance of phages and the emergence of mutations at a high rate (essentially single nucleotide or short deletions). Different genes involved in synthesis of phage receptors, mostly lipopolysaccharide (LPS) and type IV pili were affected, and we showed that phase variation was a major factor in inducing mutations [16]. Pseudolysogeny has been observed in different phage/bacteria systems in slowly growing or starved cells and is believed to be a mean to preserve phage and bacteria until better conditions are available [17]. In *Propionibacterium acnes*, establishment of pseudolysogeny by phages (all belonging to a single genus) is highly dependent on the host strain [18,19]. It is likely that such long-term maintenance of phages, which may be induced by lack of resources or by specific bacterial associations, such as in communities and biofilms, may be frequent in nature, thus favoring bacterial evolution.

Here we selected a new clinical *P*. *aeruginosa* strain, PcyII-10, susceptible to numerous phages, to further investigate the emergence of pseudolysogeny in response to infection by a lytic phage and the nature of resistant variants. We confirmed the importance of pseudolysogeny as a natural state of lytic phage/host interaction but in addition to our previous work with PAO1, we observed a high level of large chromosomal deletions arising during phage-maintenance, some being unrelated to phage resistance. The results also suggest that two unsuspected bacterial genes could be involved in phage resistance.

## Material and methods

### Ethics statement

The present project is in compliance with the Helsinki Declaration (Ethical Principles for Medical Research Involving Human Subjects). The bacterial strain was collected as part of the patients' usual care, without any additional sampling, as previously reported [20]. Ethic committees in each hospital providing bacterial isolates were consulted and they declared that patient informed consent was not needed: the Percy hospital “Comité d’éthique et des experimentations” and the Armand Trousseau hospital “Comité Consultatif pour la Protection des Personnes dans la Recherche Biomédicale (CCPPRB) Ile-De-France–Paris–Saint Antoine”.

### Bacteria and phages

After the first steps of purification from a clinical sample, *P*. *aeruginosa* PcyII-10 strain (serotype O6) has been maintained in the laboratory for several years without colony reisolation. Its genome has been fully sequenced using PacBio and Illumina sequencing (sequence accession number LT673656) (Pourcel et al. manuscript in preparation). For the present study a single colony was picked, grown in LB and used for whole DNA genome extraction, for isolation of phage resistant variants and to prepare a stock at -80°C. The genome was resequenced (Illumina sequencing). Reference strain PAO1 was used to evaluate the number of variants resisting infection by Ab09. Strains C2-10 and PARD80 were previously characterized *P*. *aeruginosa* clinical strains [21,22].

Podovirus vB_PaeP_C2-10_Ab09 (Ab09) and myovirus vB_PaeM_PAO1_Ab27 (Ab27) were previously described [23]. Phage Ab27 was used to test the host-range of variants resisting Ab09.

### Recovery of phage-resistant variants

Two “multiplicity of infection” (MOI) of 0.001 and 0.01 were used to infect PcyII-10 or PAO1 and recover resistant variants, as previously described [16,24]. Bacteria surviving infection after 72 h at 37°C were purified by three passages from a colony (passage 3 or P3) before being further characterized.

### Electron Microscopy (EM) observation

To search for traces of phage multiplication in pseudolysogens the bacteria were embedded in Epon resin and sliced as described [25]. Briefly, bacteria were grown overnight from a single colony in 2 ml of LB and the bacterial pellet was recovered after centrifugation. After fixation using 2.5% glutaraldehyde, the cells were dehydrated with ethanol and acetone before being fixed in Epon “Agar low viscosity” (Oxford Instruments). The resin blocs were sliced into 90 nm sections. Before EM observation, staining was performed using uranyl acetate 2% for 15 min, followed by three 5 min washes with water.

### DNA extraction, PCR and sequencing

Thermolysates were produced by diluting 10 μl of overnight culture in 200 μl of water, heating at 95°C for 5 min and cooling on ice. For DNA purification, bacteria were lysed in lysis buffer (Tris 10 mM, pH 7.8, EDTA 10 mM, NaCl 10 mM, SDS 0.5%wt/vol), treated with proteinase K at 50 μg ml^-1^ for 2 h at 50°C, followed by one phenol and one chloroform extraction, and ethanol precipitation. PCR was performed on thermolysates or purified DNA using oligonucleotides listed in S1 Table. The expected sizes of the PCR fragment following amplification of deleted regions were: Δ02 173bp, Δ03 127bp, Δ06 260bp, Δ07 221bp, Δ14_1 171bp, Δ14_2 220bp, Δ15 191bp, Δ16 257bp, Δ17 177bp, Δ18 138bp. Sanger sequencing of PCR products was performed by Genewiz (Takeley, UK).

### Gene cloning and expression

PCR amplicons were cloned into the pUCP24 plasmid, a generous gift of Dr. Schweizer, modified in order to introduce a NcoI restriction site [26]. This is a shuttle vector which replicates in *E*. *coli* and in *P*. *aeruginosa*, and contains a multiple cloning site downstream lacZα. The PcyII-10 *rpsB* gene and ORF1587 were PCR-amplified using oligonucleotides which included restriction sites for respectively BspLU11I and HindIII, and BspHI and HindIII (S1 Table*)*. The amplicons were digested, ligated into the vector treated by NcoI and HindIII, and transformed into *E*. *coli* in which replication of pUCP24 is optimal [26]. The insert was Sanger-sequenced in a selected recombinant which was then used to transform *P*. *aeruginosa* variants by electroporation using the fast protocol described by Choi et al. [27]. Transformants were selected using 10 μg ml^-1^ Gentamycin. The colony aspect was observed under the microscope. The transformants were then tested for their susceptibility to Ab09 and its large plaque variant and to Ab27.

### Whole genome sequencing

Ten μg of purified DNA was sent for draft whole genome Illumina MiSeq sequencing to the High-throughput Sequencing Platform of I2BC (CNRS, Gif sur Yvette, France). Libraries were made from sheared fragments of DNA with a mean size of 900bp, and 250bp paired-end reads were produced. One and a half million up to 2.5 million reads were obtained corresponding to an average 130 to 230 fold coverage. The mutations were identified by comparison with the parental genome (PcyII-10 or Ab09) using tools in GeneiousR11 (Biomatters, New Zealand) as described [16].

### Nucleotide sequence accession number

The DNA sequence of the PcyII-10 strain has been deposited under accession number LT673656. The twelve archives of sequencing reads produced in the course of this study were deposited in the European Nucleotide Archive (ENA) under bioproject PRJEB18612 (https://www.ebi.ac.uk/ena/data/view/PRJEB18612; PcyII-10 resequenced colony (POU32), variants V02 (POU35), V03 (POU33), V06 (POU43), V07 (POU44), V10 (POU45), V14 (POU34), V15 (POU36), V16 (POU29), V17 (POU23), V18 (POU27), V19 (POU25).

## Results

### High frequency of deletions in PcyII-10 variants resisting phage Ab09

**Fig 1:**
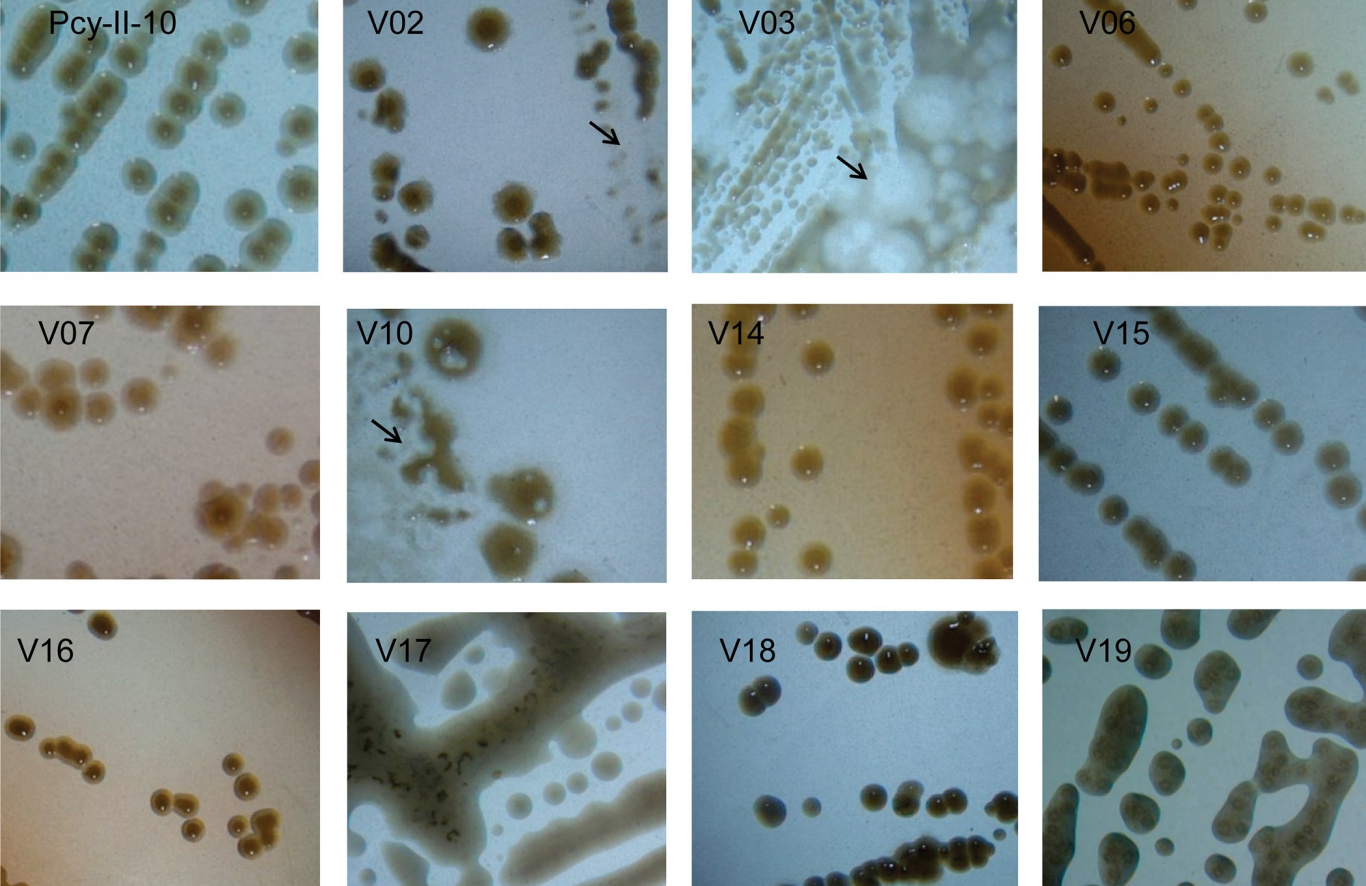
Colony morphology of PcyII-10 and phage-resistant variants. Colonies formed by PcyII-10 and eleven variants plated at P3 on LB agar were photographed after 24 h at 37°C. The arrows point to lysis zones caused by phages. Variant V03 produces small colonies with irregular shapes. Variants V06, V07, V14 and V16 produce a reddish-brown pigment, the pyomelanin. Variants V17 and V19 were mucoid.

In our previous investigations we found that phage Ab09, a N4-like podovirus, used LPS as a primary receptor but was capable of infecting PAO1 mutants defective in O-Ag synthesis (although with a lower efficiency) suggesting the existence of another receptor at the cell surface [16,25]. This phage selected for mucoid mutants with a high efficiency and was capable of establishing a pseudolysogenic state. Here *P*. *aeruginosa* PcyII-10 and PAO1 were infected by Ab09 at a multiplicity of infection (MOI) of 0.001 or 0.01 before plating the mixture in soft agar on Luria-Bertani (LB) solid medium and incubating for 72h at 37°C. Interestingly, the proportion of slow-growers among PcyII-10 colonies surviving infection was much higher as compared to PAO1 whereas the amount of large mucoid colonies was ten times less with PcyII-10 (S1 Fig). Twenty PcyII-10 resistant variants displaying different phenotypes, including two mucoid variants, were recovered and purified by three colony-isolation steps (passage 3 or P3). Nine variants grew very poorly and were not further analysed. At this stage an heterogeneity in colony size and morphology was observed for many variants, a characteristic reminiscent of PAO1 pseudolysogens behavior (Fig 1) [16]. On LB solid medium, lysis could be seen in densely rich bacterial zones and in some colonies (see for example PcyII-10-V02, PcyII-10-V03 and PcyII-10-V10 in Fig 1), most likely caused by the release of phages. We tested for the presence of phages in the supernatant of P3 overnight cultures in LB and found titers ranging from 10^7^ to 3x10^10^ phages per ml in all except PcyII-10-V14 and PcyII-10-V17. Four variants (PcyII-10-V06, PcyII-10-V07, PcyII-10-V14 and PcyII-10-V16) formed small reddish-brown colonies whereas both mucoid and non-mucoid colonies were produced by variant PcyII-10-V17. Finally, eleven variants were kept at P3 for storage at -80°C in glycerol, whole genome sequence (WGS) analysis and phenotypic characterization (Fig 1).

### Persistence of the phage

**Fig 2:**
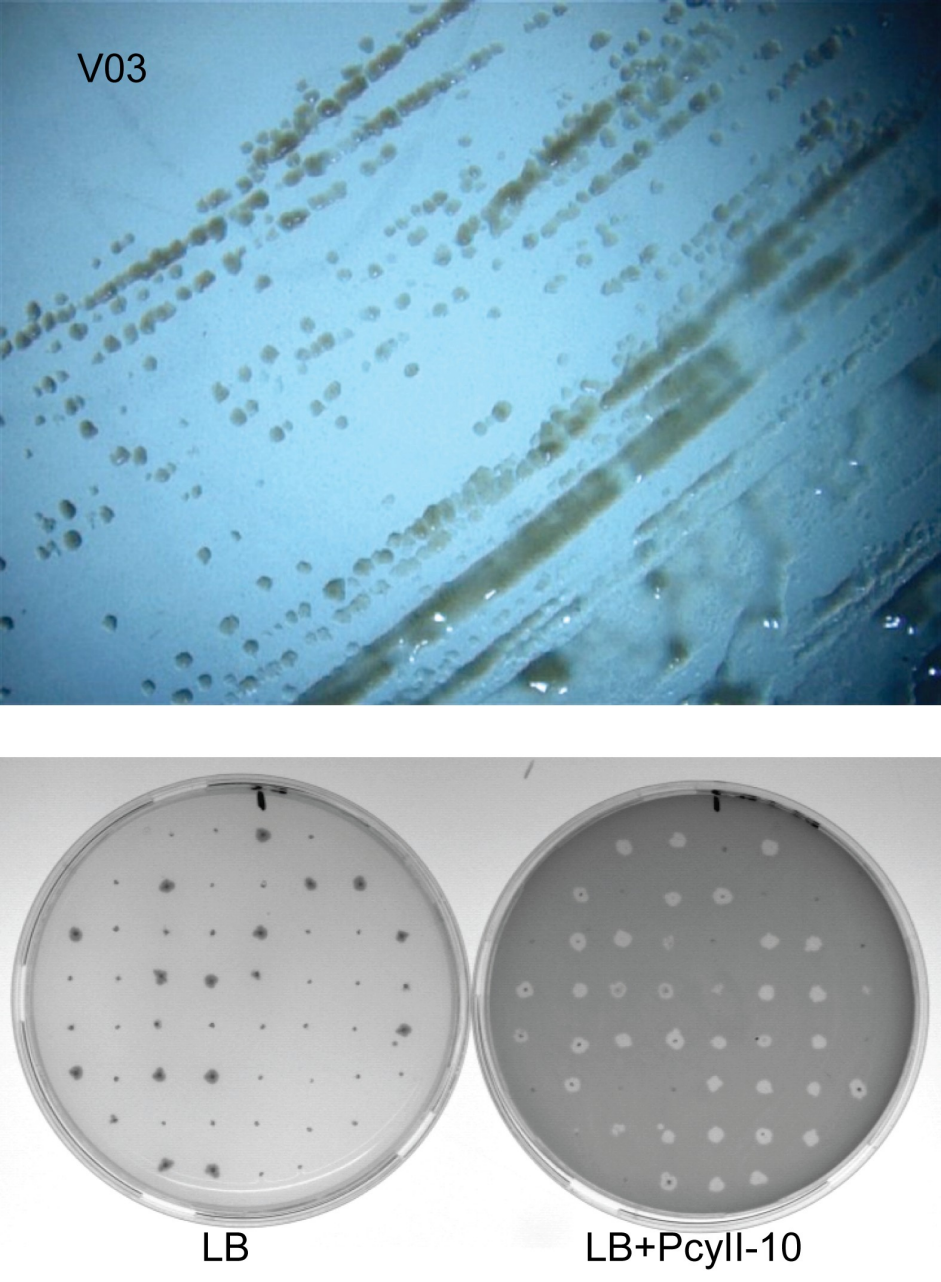
Evaluation of Ab09 persistence in PcyII-10-V03. (A) Colonies obtained after plating the P3 stock on a LB agar plate. (B) Fifty-two colonies recovered from the P3 stock were simultaneously plated using a sterilized pipette tip on LB agar (left) and on PcyII-10 embedded in soft agar overlay (right). The clear zone around the bacterial colony in the right panel is due to phage lysis of the indicator bacteria.

We analysed the capacity of five variants to produce phages after additional replatings by spreading the P3 stock on LB agar plates (Fig 2 top panel shows PcyII-10-V03) and inoculating 52 colonies first on an LB agar plate then on a plate covered with a lawn of soft agar containing PcyII-10 (Fig 2 bottom panel) [24]. A halo of lysis was observed around bacteria producing phages and these grew poorly on the LB plates. The percentage of P4 colonies producing phages was 40, 73, 20, 50, 80% for PcyII-10-V02, PcyII-10-V03, PcyII-10-V07, PcyII-10-V08 and PcyII-10-V10 respectively. The procedure was repeated eight times with variant PcyII-10-V03, each time selecting phage-producing bacteria and we observed that 80% of the colonies were still pseudolysogens. Occasionally larger colonies could be observed, suggesting the emergence of sub-variants as previously observed in PAO1.

**Fig 3:**
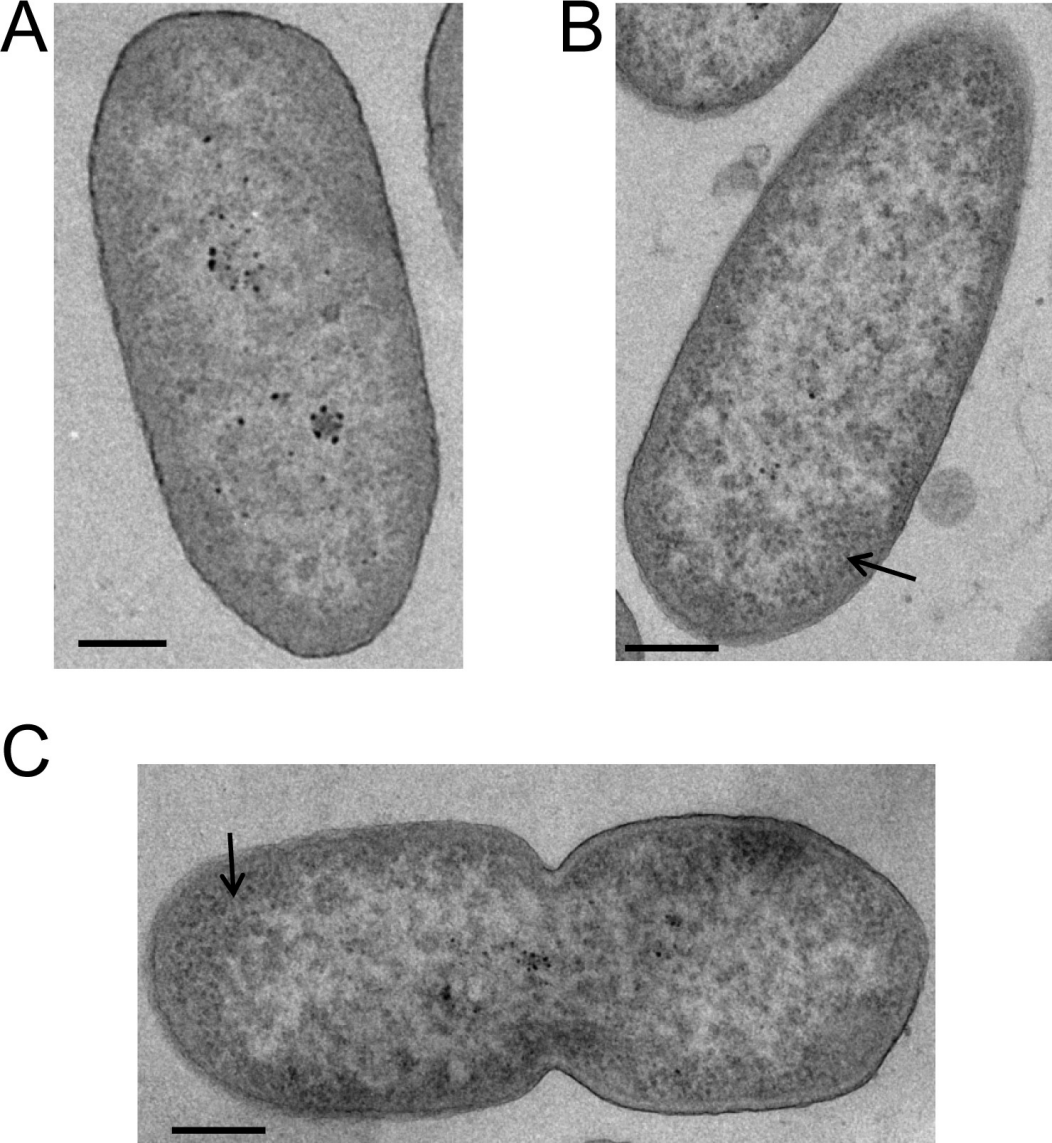
Observation of pseudolysogens by electron microscopy. Transmission electron micrograph of bacterial section from (A) The parental strain PcyII-10, (B) Variant PcyII-10-V07 and (C) Variant PcyII-10-V10. The arrow in (B) and (C) points to tightly packed material that might correspond to ribonucleoprotein particles. The scale bar corresponds to 200nm.

To search for the possible presence of complete virions inside pseudolysogens, variants PcyII-10-V07 and PcyII-10-V10 at P3 were cultured in LB, embedded in resin before thin sections were analysed by electron microscopy (EM). No evidence of virions could be found inside the bacteria but densely stained and tightly packed structures could be observed in the two pseudolysogens. They may represent ribonucleoproteins (RNPs) as previously described in bacteriophage infected cells [28,29], suggesting the existence of active metabolism. A large number of PcyII-10-V07 cells had clear cytoplasm and altered membranes, some showing blebs, a sign of cellular death (Fig 3 shows PcyII-10 and the variants PcyII-10-V07 and PcyII-10-V10). This suggested that there was a block in the multiplication cycle, probably at the late stage before production of virions. The observed structural alteration might be the result of the mutations as well as that of phage multiplication.

### Whole genome sequencing of Ab09-resistant variants

**Fig 4:**
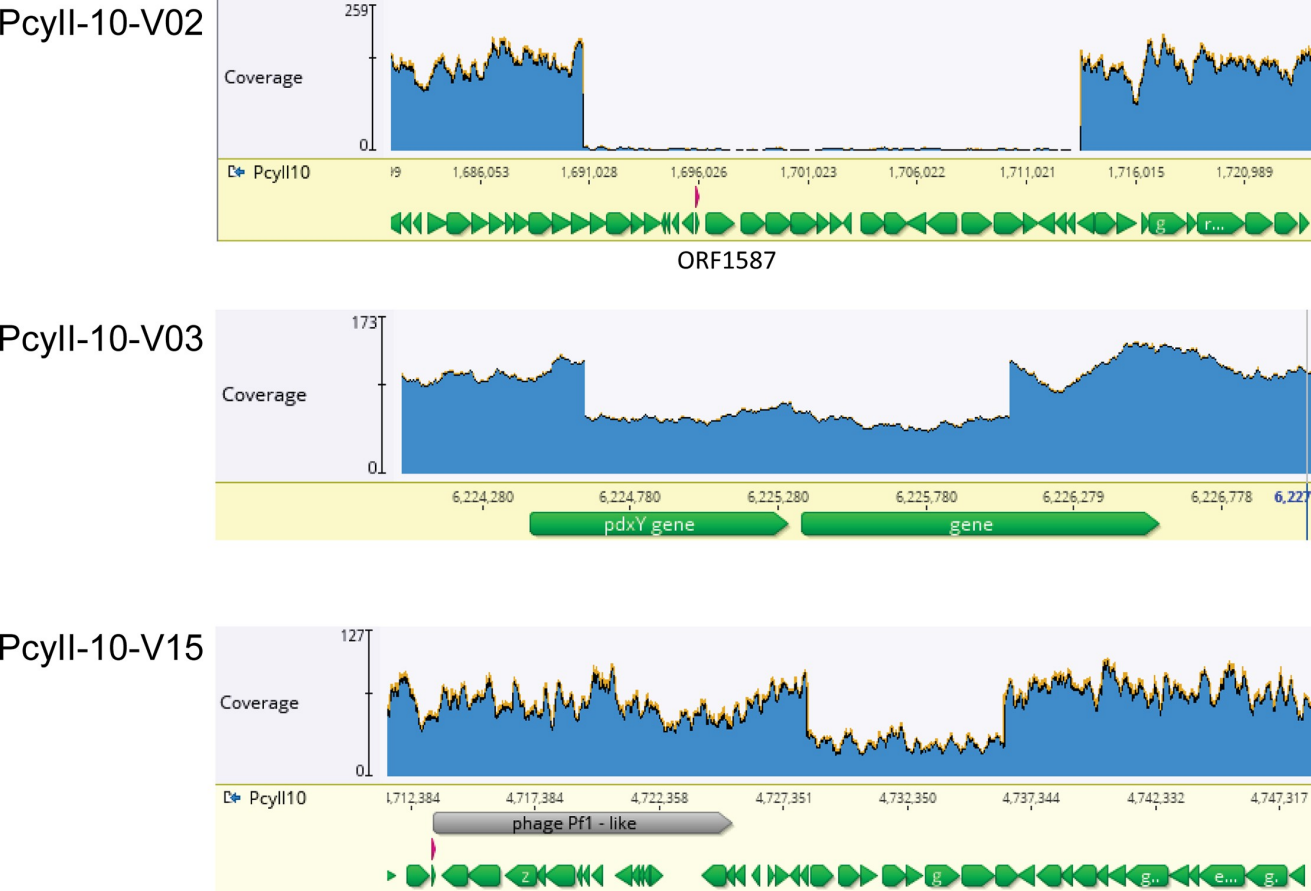
Distribution of sequencing reads on the PcyII-10 genome forthree Ab09-resistant variants. Mapping of sequencing reads to the genome of PcyII-10 revealed the existence of deleted zones. In variant PcyII-10-V02 no reads mapped to the Δ02 region, whereas in PcyII-10-V03 and PcyII-10-V15 the Δ03 and Δ15 regions were undeleted in an estimated 50% and 40% of cells respectively.

The genome of eleven Ab09-resistant variants at P3 was totally sequenced as well as that of the parental PcyII-10 culture allowing the identification in the variants of five single nucleotide changes and ten deletions (Table 1). Two variants possessed only a single nucleotide mutation (PcyII-10-V10 and PcyII-10-V19), four possessed a single deletion (PcyII-10-V02, PcyII-10-V06, PcyII-10-V16 and PcyII-10-V18) and five were double mutants. As revealed by the mapping of sequencing reads on the progenitor PcyII-10 genome, the mutations were not always present in 100% of the cells (Table 1). Whereas in three samples the whole population of cells appeared to possess a deletion, in six variants, one or two deletions were present only in a fraction of cells (Fig 4 shows three examples). Point mutations were always detected in close to 100% of the sequencing reads and in three variants a deletion was also detected in a fraction of reads (PcyII-10-V03, PcyII-10-V15, PcyII-10-V17). Four single nucleotide changes occurred in a mononucleotide stretch, causing phase-variation mutations. The existence of a mutation and/or deletion in only a fraction of the cells and the high frequency of double mutants even after three colony-purification steps suggested that new genetic modifications emerged inside pseudolysogenic bacteria, the situation previously observed with PAO1 resistant to lytic phages. The striking difference with PAO1 phage-resistant variants was the existence in PcyII-10 variants of a variety of deletions never observed in PAO1, some being very large. Ab09 reads were found in all sequenced samples, reaching relatively high levels (above 0.6%) in four variants.

**Table 1:** Characteristics of the mutations in the eleven phage resistant PcyII-10 variants.

Variant (P3)	Δ	Deletion (bp)	Positions		Read %	Point mutation	Position	Read %	Phenotype	Ab09 DNA %
V02	02	22,763	1,690,775	1,713,537	100%	** **		** **	** **	0.6
V03	03	1,434	6,224,639	6,226,072	50%	*rpsB* A(3)->A(2)	1,402,765	100%	Blue	1.84
V06	06	535,972	2,930,640	3,465,943	100%				Brown	
V07	07	276,763	3,053,155	3,334,409	80%				Brown	
V10						*ORF1587* T(4)->T(3)	1,697,201	100%		0.19
V14	14_1	2,999	1,941,686	1,944,684	20%					3.69
	14_2	56,271	3,273,717	3,329,987	50%				Brown	
V15	15	7,920	4,728,358	4,736,277	40%	*ORF1587* T(7)->T(8)	1,697,063	95%		0.66
V16	16	340,123	3,052,982	3,393,104	100%				Brown	
V17	17	11,975	4,713,278	4,725,252	80%	*mucA* C(2)->C(1)	4,678,812	100%	Mucoid	
V18	18	64,894	4,713,278	4,778,171	50%					
V19						*mucA* G->A	4,678,7,60	100%	Mucoid	

### Identification of the mutations and relationship with phage resistance

**Fig 5:**
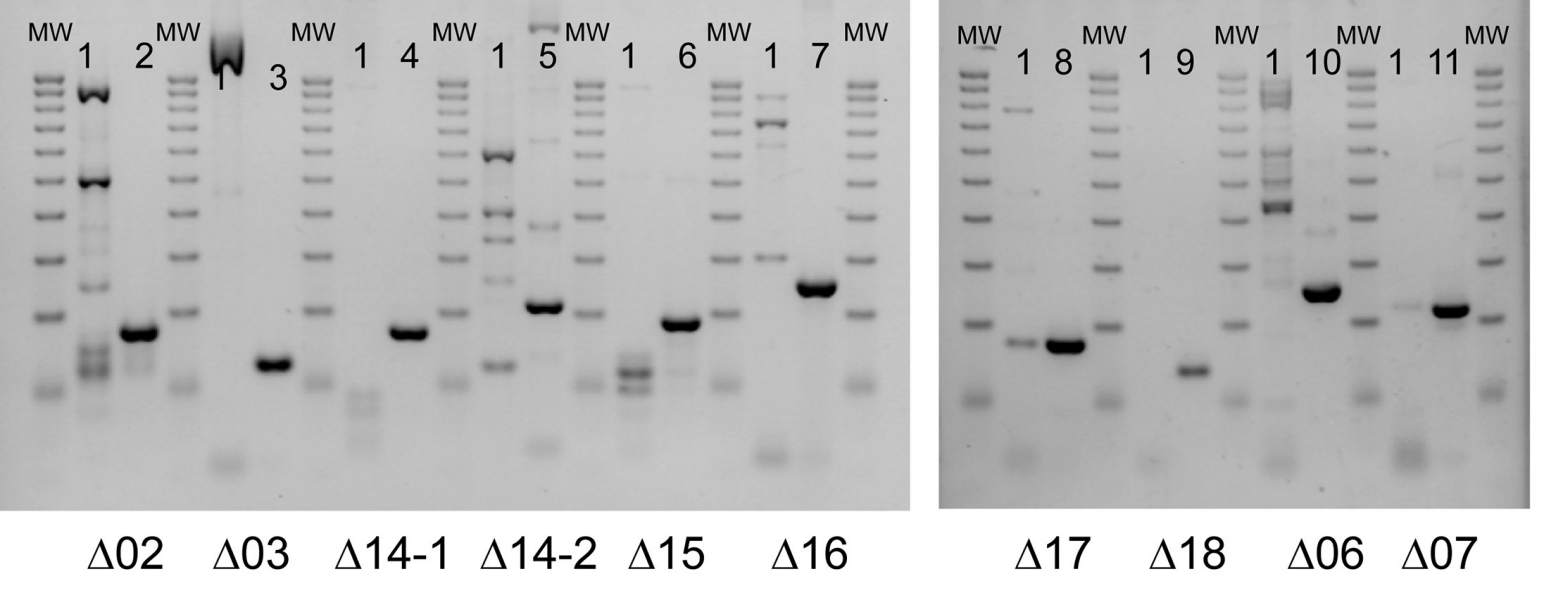
Analysis of ten deletions by PCR amplification and agarose gel electrophoresis. For each deletion, two DNA samples were analysed, PcyII-10 and the corresponding variant. MW is for Molecular Weight marker, a 100bp fragment ruler from 100bp to 1000bp.

Because sequencing revealed the existence of mixed populations of cells in some variants at P3, we performed further colony purifications from frozen stocks and searched for the presence of the detected single mutations or deletions by PCR analysis and Sanger sequencing. Using primers selected on both sides of the deleted region and inside this region (S1 Table) all the deletions could be confirmed by PCR analysis, on the DNA used for whole genome sequencing and in newly isolated colonies (Fig 5). Similarly, the point mutations were verified by PCR amplification and direct Sanger sequencing of the amplification product, confirming their absence at a detectable level in PcyII-10 before infection, and presence in the variants. We also checked for the presence of Ab09 and selected colonies that were free of phages in order to analyse the effect of the sole mutations. Variants were tested for their susceptibility to Ab09 and to the *P*. *aeruginosa* myovirus Ab27 which uses LPS as a primary receptor [25] (Table 2).

**Table 2:** Susceptibility of the PcyII-10 variants and subvariants to phages.

Phage-free isolates	Deletion (Δ)^a^	Mutation	Phenotype^b^	Phage susceptibility^c^	
				Ab09	Ab27
V02	02		UN	S	R
V03(1)	wt	*rpsB*	Blue	R	S
V03(2)	03	*rpsB*	Blue	R	S
V06	06		SC brown	R	R
V07(1)	wt		LC pale	S	R
V07(3)	07		SC brown	R	R
V10(1)		wt	UN	S	S
V10(4)		ORF1587	UN	R	R
V14(3)	14_1		UN	I	I
V14(8)	14_2		SC brown	R	R
V15(1)	15	ORF1587	UN	R	R
V15(7)	wt	ORF1587	UN	R	R
V16	16		SC brown	R	R
V17-nmuc^d^	17(Pf1)		UN	S	S
V17-muc^d^	17(Pf1)	*mucA*	Mucoid	I	R
V18(5)	18		SC	R	R
V18(7)	wt		UN	S	S
V19		*mucA*	Mucoid	I	I

^a^ wt, wild type

^b^ UN, unaffected, SC, small colonies; LC, large colonies

^c^ S susceptible, R resistant, I intermediate growth

^d^nmuc, non mucoid, muc, mucoid

**Fig 6:**
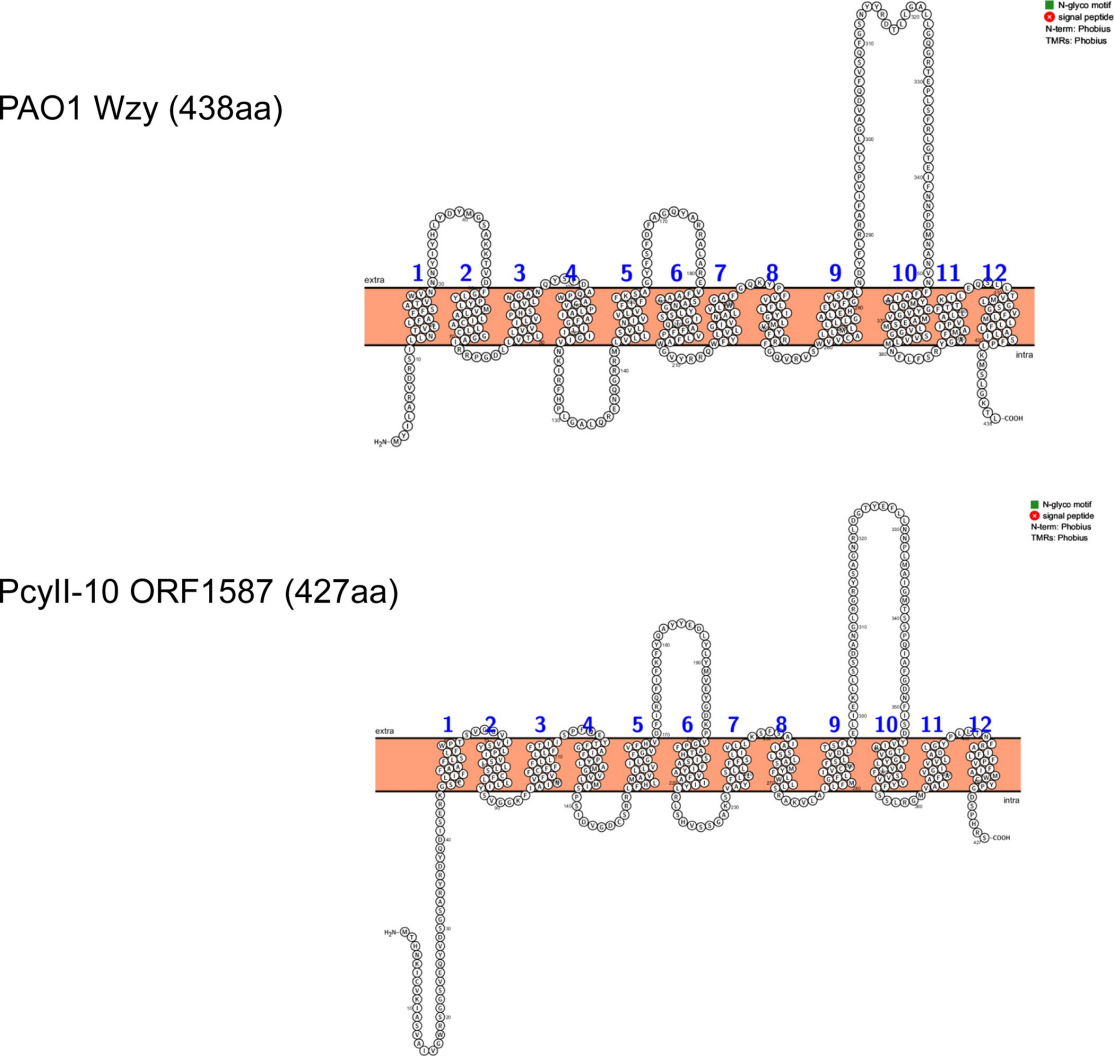
Comparison of Wzy and ORF1587 topologies using Protter. (http://wlab.ethz.ch/protter/start/).

As expected, all the tested colonies from PcyII-10-V02 possessed the 22,763bp deletion Δ02 which encompassed the aliphatic amidase operon amiEBCRS involved in motility and virulence [30], genes of the carbon-phosphorus lyase pathway and others involved in synthesis of the flagella. Notably, Δ02 included ORF1587 encoding a Wzy-like membrane protein, and found to be mutated in two other variants, PcyII-10-V10 and PcyII-10-V15. ORF1587 shows important similarities at the structural level with Wzy an oligosaccharide polymerase involved in O-Ag B-chains biosynthesis of certain serotypes, such as PAO1 O5 (Fig 6). A PcyII-10-V02 colony free of phages was susceptible to phage Ab09 and totally resistant to Ab27. Thus in PcyII-10-V02 we suppose that primary phage-resistance to Ab09 was due to immunity conferred by the presence of phage Ab09, still detected after three steps of colony purification (0.6% of reads), and not to deletion Δ02.

The sequenced PcyII-10-V03 genome showed both a mutation in *rpsB*, the gene for the 30S ribosomal protein S2, and a 1,434bp deletion (deletion Δ03). The growth of variant V03 was very poor and at P3 it still produced phages at high concentration. The *rpsB* mutation, an A(3)->A(2) single nucleotide deletion present in 100% sequencing reads which resulted in premature stop and production of a 134 amino acids protein, could be confirmed by PCR amplification and Sanger sequencing. The *rpsB* gene is an essential component of the translation machinery in *Escherichia coli* [31]. Deletion Δ03 which encompassed two genes, *pdxY* a pyridoxal kinase and an ORF encoding an acyltransferase of unknown function, was originally present in half of the bacterial population, as shown by the distribution of sequencing reads. Out of eight colonies obtained from the frozen P3 stock, four possessed the deletion. Non-deleted PcyII-10-V03 bacteria such as variant PcyII-10-V03(1) were resistant to Ab09 suggesting that the *rpsB* mutation was sufficient to confer resistance to Ab09. Interestingly, phage Ab27 could grow normally on this mutant.

All the PcyII-10-V06 colonies were small, reddish-brown, and possessed a single large deletion of 536kb (Δ06) including the homogentisate 1,2-dioxygenase *hmgA* gene, the absence of which is responsible for the brown color [32], and *galU* a key gene for the O-Ag biosynthesis. This variant was resistant to Ab09 and Ab27.

Red and white colonies were observed upon replating of the PcyII-10-V07 P3 stock, the red ones being smaller in size and possessing the 277kb deletion Δ07 which encompassed *hmgA* and *galU*. The red colonies were resistant to Ab09 and Ab27. A subvariant, PcyII-10-V07(1), forming large white colonies was not deleted and was normally susceptible to Ab09 but resistant to Ab27, suggesting the emergence of a novel mutation.

PcyII-10-V10 possessed a T(4)->T(3) mutation in ORF1587 resulting in production of a truncated 248 aminoacids protein (instead of 427aa). PcyII-10-V10 at P3 produced a large amount of phages and upon replating, different colonies could be picked with different phenotypes. PcyII-10-V10(1) forming normal size colonies was susceptible to Ab09 and Ab27 and was not mutated in ORF1587 whereas three other tested colonies of smaller size (such as V10(4)) were mutated and resisted infection by Ab09 and Ab27.

Two derivatives of PcyII-10-V14 could be obtained containing deletion Δ14_1 or deletion Δ14_2. Variant PcyII-10-V14(3) with the 2,999bp deletion Δ14_1 formed aggregates in LB and showed reduced susceptibility to Ab09 and Ab27 (small turbid plaques). Δ14_1 included four genes (ORF1811 to ORF1814): *wbpT*, *wbpU*, *wbpV* and *wbpL*, involved in O-Antigen biosynthesis [33]. PcyII-10-V14(8) with the 56,271bp deletion Δ14_2 and making red colonies, conferred resistance to Ab09 and Ab27. Δ14_2 extended from ORF3001 corresponding to gene *yejE*, to part of gene *hmgA* and included *galU*.

PcyII-10-V15 also possessed a mutation in ORF1587 (T(7)->T(8)) causing a frameshift and premature stop and production of a truncated protein of 332 amino acids. The mutation was found in all tested colonies at P4. In addition sequencing revealed a 7,920bp deletion (deletion Δ15) extending from ORF4387 (*lsrG* gene) involved in control of quorum sensing, to ORF4395. Δ15 started 3kb away from a Pf1-like prophage copy and included the *migA* gene, an alpha-1,6-rhamnosytransferase involve in LPS biosynthesis, and a membrane protein of unknown function. Among 16 tested PcyII-10-V15 colonies all resisting Ab09 and Ab27, only five possessed deletion Δ15, PcyII-10-V15(1) possessing the deletion and PcyII-10-V15(7) being intact. Analysis of ORF1587 in PcyII-10-V15(7) confirmed the presence of the mutation, suggesting that alteration of this gene was responsible for resistance.

PcyII-10-V16 colonies were small, reddish-brown, and possessed a single large deletion of 340kb (Δ16), including the *hmgA* and *galU* genes. They were fully resistant to both Ab09 and Ab27.

Deletion Δ17 in variant PcyII-10-V17 corresponding to a copy of the Pf1-like prophage was confirmed in six isolated colonies out of 10. Among them, a mucoid colony had in addition a point mutation in *mucA* conferring resistance to Ab27 and reduced growth of Ab09. Non-mucoid colonies were susceptible to both phages.

PcyII-10-V18 possessed deletion Δ18 encompassing the Pf1-like prophage and 64,894 bp flanking genomic DNA. Genes in this region included, in addition to *migA*, several glycosyltransferases, a Type II secretion system and genes for iron transport. PcyII-10-V18 was resistant to both Ab09 and Ab27. A large amount of Pf1-like reads were found among the genome sequencing data showing that Pf1 multiplied actively at the third passage in this variant.

PcyII-10-V19 colonies were all mucoid due to a mutation in *mucA* which conferred reduced susceptibility to both Ab09 and Ab27.

### Genomic distribution of deletions

The deletions were distributed into six genomic regions, three of them associated with a unique event (Δ02, Δ03, Δ14_1) and two with respectively three (region N°1) and four (region N°2) events. In region N°1 Δ17 and Δ18 included the Pf1-like prophage whereas Δ15 started 3kb after this prophage insertion site suggesting a role for the virus in the deletion. Δ06, Δ07, Δ14_2 and Δ16 encompassing the *hmgA* gene covered region N°2 at the replication terminus.

We searched for the presence of direct repeats flanking the deleted regions and found them in Δ02, Δ03, Δ06, Δ14_1 and Δ18, with length of respectively eight nucleotides (5’-CGGCGCCA-3’), nine nucleotides (5’-CGCTGGTGG-3’), ten nucleotides (5’-CGCCTGCTCG-3’), ten nucleotides (5’-TGGGGGATGC-3’) and seven nucleotides (5‘-AGGGTTC-3’). No significant conserved sequences were found flanking the other deletions.

### Variability of phage genomes and isolation of large-plaque forming variants

Ab09 phage reads were found in all sequenced variants and in particularly significant amounts (0.6% to 3.69% Table 1) in PcyII-10-V02, PcyII-10-V03, PcyII-10-V14 and PcyII-10-V15, allowing assembly of a genome with a higher than 50X coverage, and search for possible mutations as compared to the parental Ab09 phage. The Ab09 phage genome assembled from PcyII-10-V02 and PcyII-10-V14 reads was identical to the parental genome. In variant PcyII-10-V03 the phage possessed a single G->T mutation at position 29,815 in ORF48 encoding a putative 1100 amino acids tail fiber protein, leading to a change of amino acid S289Y. In PcyII-10-V15 the assembled phage genome showed three modifications in ORF48, a G->T change in 100% of reads (position 29,815) and a A->G on about 50% reads at positions 30,159 leading to a V926A change, and a silent G->A mutation at position 32,564. This reflected an ongoing evolution of the phage in pseudolysogens.

**Fig 7:**
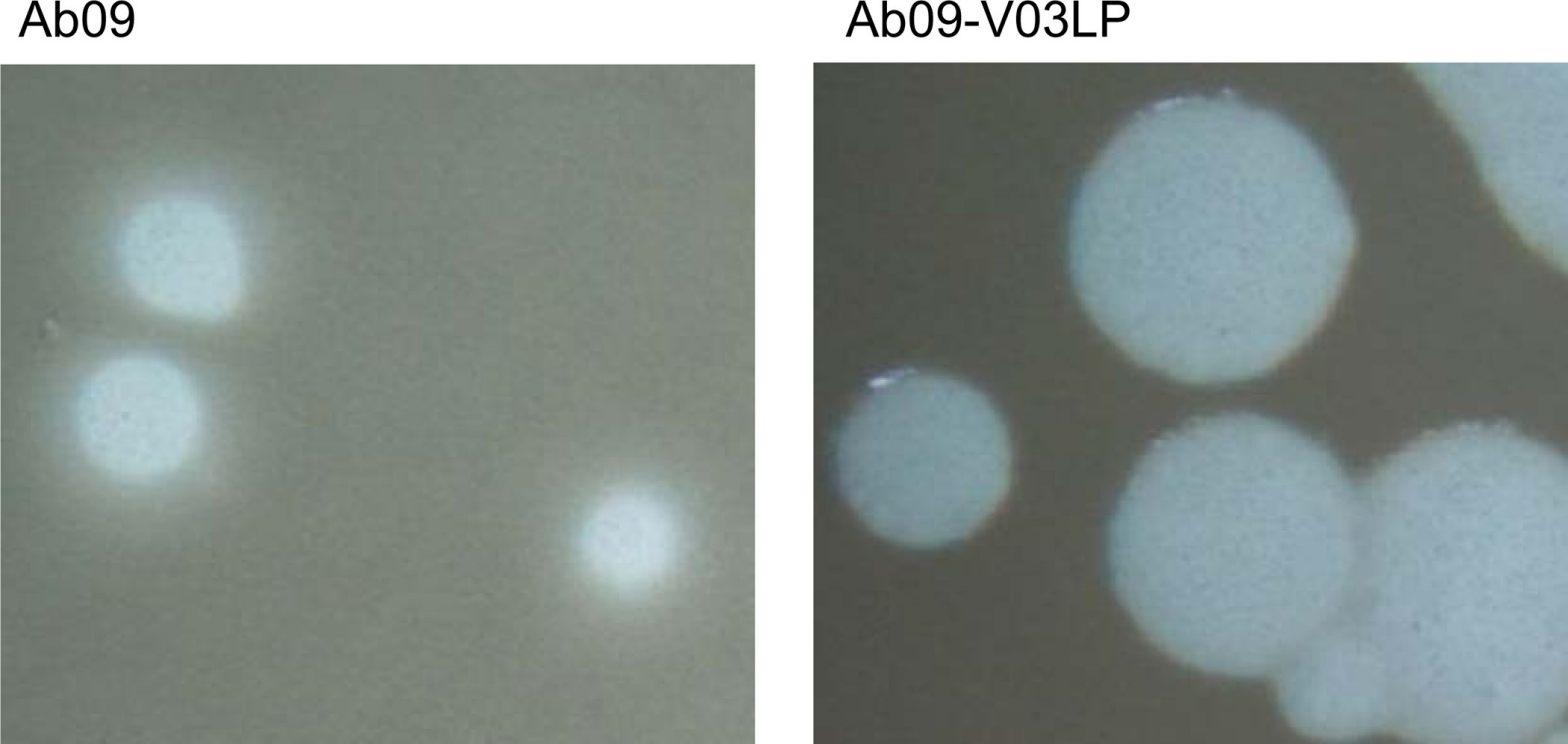
Plaque morphology of the large-plaque mutant of Ab09 produced by variant PcyII-10-V03. Plaques produced by the wild type Ab09 phage have irregular fuzzy shapes and are surrounded by a halo whereas the Ab09-V03LP mutant forms large plaques with sharp edges and no halo.

While titrating phages produced by an overnight culture of variant PcyII-10-V03 we noticed some large clear plaques among a majority of smaller plaques with a halo, the usual aspect for Ab09 plaques. We thus recovered a small (Ab09-V03SP) and a large (Ab09-V03LP) plaque, amplified the phages on PcyII-10 and performed further characterization. On PcyII-10 Ab09-V03LP plaques were 4mm in diameter, clear with sharp edges whereas the parent Ab09 and Ab09-V03SP produced 2mm plaques with irregular edges and a halo (Fig 7). On three additional strains, no difference in plaque size and morphology was seen between Ab09 and the two variants: the mean diameter was 3mm, 2mm and 4mm on *P*. *aeruginosa* strains PAO1, C2-10 and PARG80 respectively. Contrarily to progenitor Ab09 and Ab09-V03SP, Ab09-V03LP could grow on PcyII-10-V03, producing very large plaques (up to 6mm in diameter). In order to try and understand the basis for this phenotype, the complete genome of phage variant Ab09-V03LP was sequenced revealing three point mutations in ORF48. The first one in the N-terminal part, a S289Y substitution, was not specific for Ab09-V03LP as it was already present in the DNA extracted from PcyII-10-V03 pseudolysogen and also in Ab09-V03SP. The V926A substitution was observed in the phage present in the PcyII-10-V15 variant whereas the L893R substitution was specific to the large-plaque forming phage, Ab09-V03LP. This substitution in the COOH terminal region of the protein replacing an hydrophobic residue by a positively charged residue might lead to significant change in the protein secondary structure.

### The role of *rpsB* and ORF1587

Two new genes appeared to be candidates for a role in phage infection, *rpsB* and ORF1587 and we therefore performed additional experiments to test this hypothesis. We performed an adsorption assay on PcyII-10 and on isolated variants (PcyII-10-V03(1) for *rpsB*, and PcyII-10-V15(7) and PcyII-10V10(4) for ORF1587) that only possessed a mutation in these genes. On the PcyII-10 parental strain, phage adsorption was observed after 10 min whereas no adsorption was observed on the variants.

The two genes were cloned into pUCP24, an expression vector for *P*. *aeruginosa*, and the recombinants were electroporated into the mutants. Complementation of the *rpsB* mutation in PcyII-10-V03(1) resulted in normal colony phenotype and was fully efficient in restoring susceptibility to Ab09 (S2 Fig). Interestingly the expression of ORF1587 in PcyII-10-V10(4) restored susceptibility to Ab09, Ab09-V03LP and Ab27 but the plaques where very small, whereas the transformed PcyII-10-V15(7) cells remained resistant to both phages.

## Discussion

N4-like phages are podoviruses distributed into four genera frequently isolated from different Gram-negative species and characterized by the presence of a giant virion-associated RNA polymerase [34,35,36]. In *P*. *aeruginosa* the corresponding genus “Lit1virus” includes Lit1, PA26 and Ab09 which display a rather large host spectrum, making them interesting tools for phage therapy. They form plaques with a halo, likely related to degradation of capsular exopolysaccharides by a depolymerase activity [37,38,39]. The interactions between *E*. *coli* and phage N4, and the basis for phage-resistance have been largely studied but little is known about the other members of this family. We confirm here using the clinical and minimally subcultured *P*. *aeruginosa* strain PcyII-10 that Ab09 adopts a pseudolysogenic behavior at a high rate in starving bacteria, and that this favors the emergence of mutants as previously shown using the PAO1 laboratory strain [16]. During maintenance of the phage a small proportion of bacteria can support a full multiplication cycle and die releasing new phages, whereas some cells maintain small amounts of viral genome and other are cured, becoming prey for the free phages. As opposed to the carrier state established by the levivirus LeviOr01 in which large quantities of virions could be seen inside enlarged cells [40], there is apparently no Ab09 virions accumulating in the pseudolysogens, but the bacteria appear to be deeply affected. Therefore the blockage might be at the level of phage multiplication and not lysis. This was observed with phage T4 in starved cells, a phenomenon called lysis inhibition (LIN) induced by superinfection of T4-infected cells at any time during the multiplication cycle [41,42]. Some core and variable genes in members of the N4-like family show striking similarities to those of the T4 superfamily [43]. Phage Ab09 possesses two genes related to phage T4 rIIA and rIIB which appear to play an indirect role in LIN, promoting delayed lysis but there is apparently no Ab09 equivalent of gene rI which is the real actor of LIN [44,45,46].

In this work we show that the fate of infection by phage Ab09 is dependent on the host. In PcyII-10 variants resisting Ab09 infection point mutations are rather rare as compared to PAO1 phage-resistant mutants, whereas deletions are very abundant. The rate is higher than the expected *in vitro* deletion rate calculated in *Salmonella enterica* growing in rich medium [47] and some of these deletions are very large. In addition, in a majority of variants the deleted genomes are found in less than 100% of cells after three steps of colony isolation and could be segregated by further subplatings. This shows that the deletions took place after the initial purification of the phage-resistant colonies and therefore in bacteria which continued to evolve. Curiously, some of the deletions appear not to be necessary for resistance to Ab09. We hypothesize that persistence of Ab09 in the bacteria in a pseudolysogenic state destabilizes the genome, leading to an important degree of secondary deletions.

Two deletions involve a copy of the Pf1-like prophage, and free Pf1-like phage DNA is detected in significant amounts in three additional resistant variants, suggesting that Ab09 infection leads to the prophage activation. In addition to Pf1 prophage excision, flanking chromosomal DNA is deleted in PcyII-10-V18 and the deletion in PcyII-10-V15 was 3kb away from the insertion site, suggesting that this region is a hotspot for chromosome rearrangement. The presence of a Pf1-like filamentous phage was previously proposed to be associated with the production of small colony variants (SCVs) [48]. PcyII-10 spontaneously produces SCVs but the link with Pf1-like phage activation is not established. Activation of Pf1 transcription was observed in bacteria infected with phage phiKZ although the amount of Pf1 DNA was not increased, and it was assumed that the filamentous phage was unsuccessfully attempting to escape the cells [49].

Four different deletions were found in a region (called here region N\2) in which other large deletions were already described in association with a brown/red pigmentation. This was the case for example in *P*. *aeruginosa* strains isolated in the course of an eight-years survey of a single cystic fibrosis (CF) patient (188kb deletion) [50], in *P*. *aeruginosa* CF strains [51] and in *P*. *aeruginosa* strain P1A resistant to PAP1, a PAKP1-like phage [52]. All the deletions affect *galU*, a key gene in LPS biosynthesis, most likely responsible for the phage-resistance. Pyomelanin production responsible for the red color is due to loss of the homogentisate gene *hmgA* (position 3,329,705 to 3,331,003) [32,53]. This loss was reported to favour persistence in the lung of CF patients [32], and to increase stress resistance, persistence, and virulence in some bacterial species [54,55], but it can also produce an opposite effect in other specie [56]. The regions covered by the large deletions are centered on the replication terminus. It was found that pausing of DNA replication was a factor favoring deletion formation in *E*. *coli* [57] and that in *Salmonella* the replication terminus is a hot-spot for transposon-mediated deletion [58].

As compared to deletions spontaneously occurring in chronic infections, we did not find a direct repeat on both side of all the large deletions containing the *hmgA* gene [55] in agreement with observations by Shen et al. [59]. Indeed these authors showed that MutL promotes large chromosomal deletions through non homologous end joining (NHEJ). Repeats were present flanking the other deletions implying a role of *recA*-mediated recombination. Illegitimate recombination was likely to be responsible for five deletions with homologous region of 6bp to 10bp in length. For bacteria it was proposed that 20 to 25bp of homologous sequence is required for RecA-dependent homologous recombination [60,61]. Mutations in MutS and SbcCD, the prokaryotic homologue of Rad50/Mre11 enhance illegitimate recombination and increase the rate of deletion [12,62]. A portion of the SbcCD subunit C gene is deleted in PcyII-10 which may have an effect on deletion rate. MutS is intact in PcyII-10 and cannot be involved in the deletion rate. We analysed additional known antimutator genes and did not find any significant differences in PcyII-10 as compared to PA14 or PAO1 reference strains [63].

All mutation/deletions except one, the *rpsB* mutant in V03, are likely to affect the biosynthesis of LPS. Mutations were found at two different positions in a gene of unknown function, ORF1587, both leading to the production of a truncated protein, lack of O-Ag B chains and resistance to Ab09 and Ab27. ORF1587 lies between the Arg tRNA and *amiE* genes, a region of heterogeneity in serotype O6 *P*. *aeruginosa* strains. As the equivalent of *wzy* was not found in the O-antigen biosynthesis gene cluster of O6 strains it is possible that ORF1587 functions like Wzy [33,64]. Pan et al. described at the same position in the genome, a gene that they believe is the *wzy* gene, Y880_03157 of O6 PAK *P*. *aeruginosa* [65]. As previously shown for Wzy proteins in different serotypes, ORF1587 encodes a protein with a similar topology but with little homology at the amino acid level (Fig 5) [64,66]. Complementation of variant PcyII-10-V15 with the wild type gene did not restore susceptibility to Ab09 and this may be due to the dominant action of the mutated gene. It is compatible with the fact that PcyII-10-V02, deleted for ORF1587 was susceptible to Ab09 and suggests that another *wzy*-like gene is present in PcyII-10.

Although carbohydrate chains of the LPS seem to be the first molecules to which the phage binds, it seems reasonable to think that a receptor at the membrane is important for secondary adsorption and ejection of the DNA as observed with other podoviruses [67]. Four gene products are required for the adsorption of *E*. *coli* N4 phage (NfrA, B, C, D), one of which is a cytoplasmic protein [68,69]. Both host and phage proteins are thought to constitute a channel spanning the outer and inner membranes through which the DNA and the virion-associated RNA polymerase are injected into the cell. The N4 protein gp65 which constitutes the sheath surrounding the tail tube is adsorbed on the cell surface by recognition of the outer membrane protein NfrA (+ NfrB) [70]. Interestingly, we observed a mutation in a gene that was not affected in PAO1 variants resistant to Ab09 and which may be specifically involved in binding of Ab09 to its receptor. Mutation in *rpsB* in variant PcyII-10-V03 leading to synthesis of a truncated protein was responsible for altered growth but did not appear to be lethal. PcyII-10-V03 resisted infection by Ab09 but was susceptible to Ab27. A derivative of Ab09 with increased growth capacity was isolated from PcyII-10-V03 P3 pseudolysogens and it showed a mutation in a putative tail fiber protein. Like other ribosomal proteins, RpsB has been shown to be exposed at the surface of *Pseudomonas* and other Gram^-^ bacteria [71,72] and also found in the periplasm [73]. It is possible that the phage fiber interacts with RpsB for infection. Alternatively protein S2 of the 30S ribosomal subunit may play a role in translation of leaderless mRNAs as demonstrated for phage lambda [74]. However our results are preliminary, and additional investigations will be necessary to understand the precise role of RpsB in phage susceptibility.

As previously observed in PAO1 pseudolysogens, long-term maintenance of phages leads to emergence of phage mutants that outgrowth the parental phage. Interestingly the observed mutants are all tail fiber protein mutants. At P3, the Ab09-V03 phage variant produced two kinds of plaques, one of which was reminiscent of the large sharp-edged plaques (r-type) formed by T4 phages R rapid-lysis mutants, as compared to the wild type rough-edged plaques [75,76]. Sequencing of the large-plaque forming phage showed mutations in the putative tail fiber gene ORF48 a protein which in similar phages form a trimer and interacts with other tail protein and with carbohydrates. The morphology of phage plaque is the result of different factors, including adsorption and diffusion in the agar, but also efficient lysis [77]. The sharp-edged phenotype of Ab09-V03LP is apparently not related to lysis but rather to adsorption and is observed on PcyII-10 in which the phage evolved but not on three other susceptible strains. As demonstrated in a joint theoretical-experimental approach using *E*. *coli* and one of its phage, slow adsorption can maximize plaque size [78].

## Supporting information

S1 Fig *Pseudomonas aeruginosa* PAO1 or PcyII-10 colonies resistant to Ab09 phage infection. (TIF)

S2 Fig Complementation of the mutations in three variants. PcyII-10-V03(1) PcyII-10-V10(4) and PcyII-10-V15(7) were transformed either by the vector pUCP24 (A) or by the recombinants (B). Colonies were observed on medium containing Gentamycin and the bacteria were tested for their susceptibility to four dilutions of phage Ab09 (1), Ab09-V03LP (2) and Ab27 (3).
(TIF)

S1 Table Primers used for PCR amplification. (DOCX)
