# Supplementary figures and images for: Investigation of Pseudomonas aeruginosa strain PcyII-10 variants resisting infection by N4-like phage Ab09 in search for genes involved in phage adsorption
Source: PLoS One. 2019 Apr 16;14(4):e0215456. doi: 10.1371/journal.pone.0215456 (PMC6467409; doi:10.1371/journal.pone.0215456)

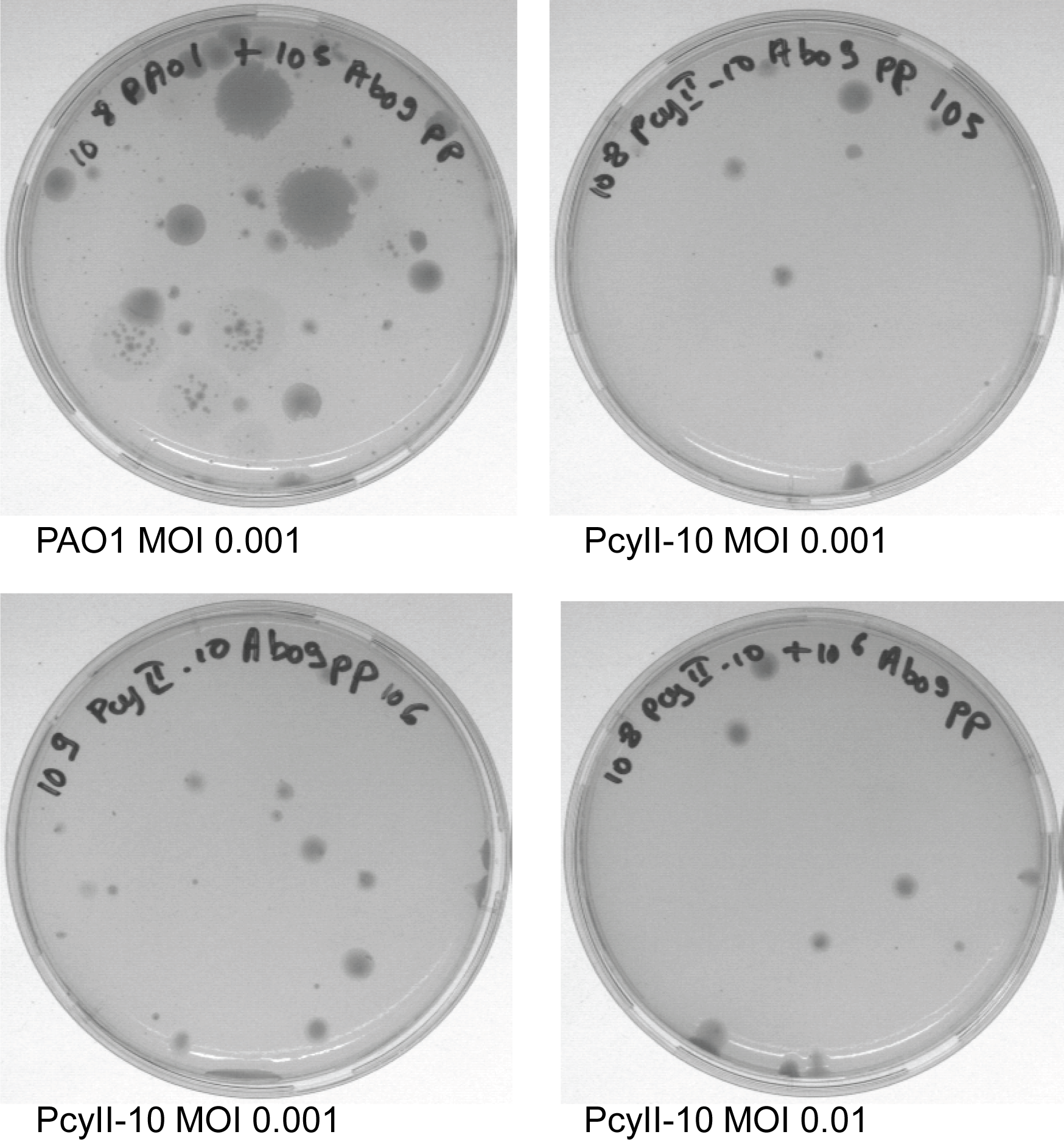

Supplement: S1 Fig — (TIF) [file pone.0215456.s001.tif]

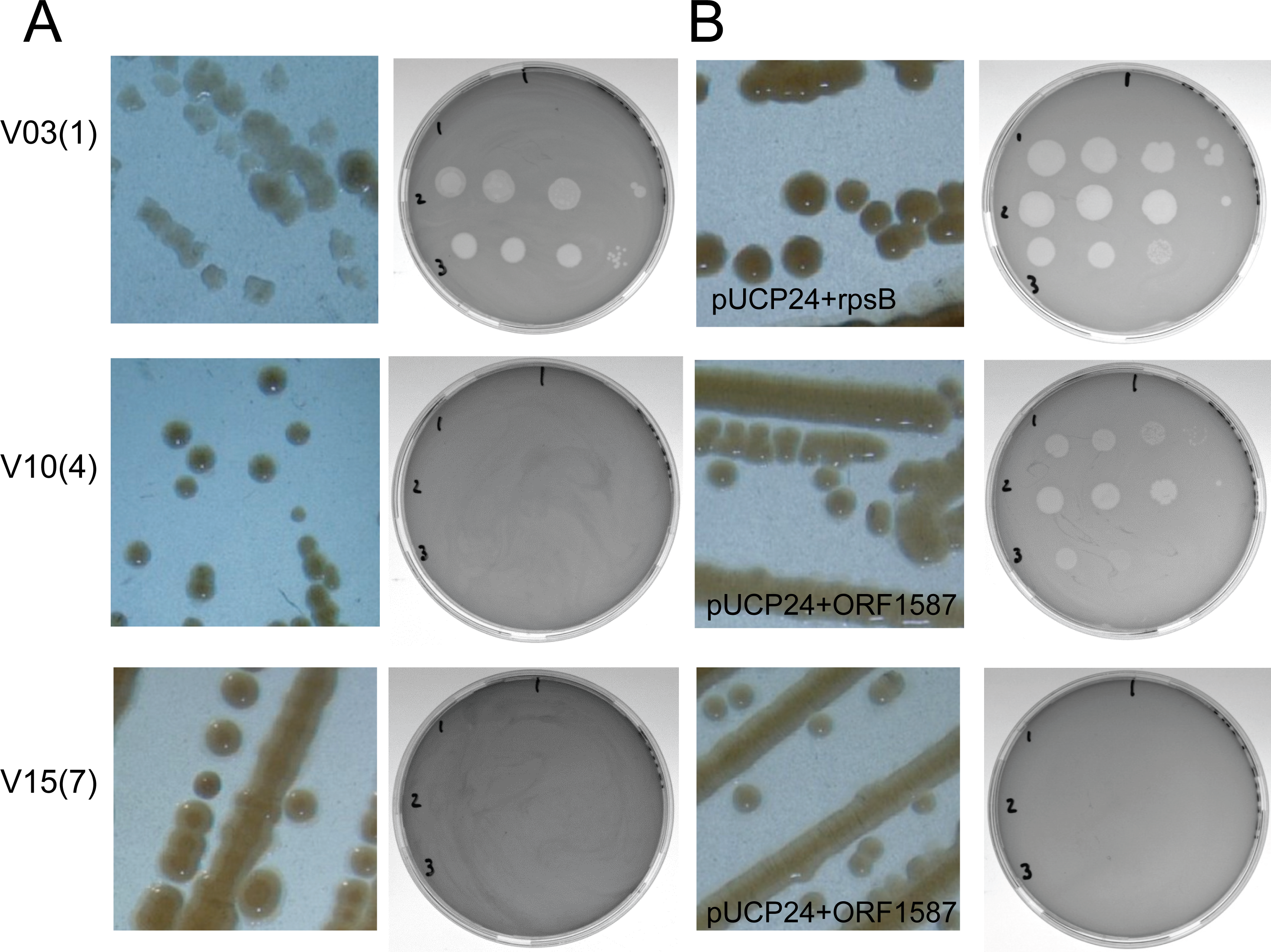

Supplement: S2 Fig — PcyII-10-V03(1) PcyII-10-V10(4) and PcyII-10-V15(7) were transformed either by the vector pUCP24 (A) or by the recombinants (B). Colonies were observed on medium containing Gentamycin and the bacteria were tested for their susceptibility to four dilutions of phage Ab09 (1), Ab09-V03LP (2) and Ab27 (3). (TIF) [file pone.0215456.s002.tif]
