# Supplementary material for: Investigation of Pseudomonas aeruginosa strain PcyII-10 variants resisting infection by N4-like phage Ab09 in search for genes involved in phage adsorption
Source: PLoS One. 2019 Apr 16;14(4):e0215456. doi: 10.1371/journal.pone.0215456 (PMC6467409; doi:10.1371/journal.pone.0215456)
Supplement: S1 Table — (DOCX) [file pone.0215456.s003.docx]

**S1 Table: Primers used for PCR amplification.**

| Primers | Sequence |
| --- | --- |
| Δ2_ext_F | CAGGTGGAGATCGATCCGCT |
| Δ2_ext_R | CCCAGGGCTACCTGACCGAT |
| Δ2_int_F | CCGACTTCCGCGAGAAATCG |
| Δ2_int_R | GCAGGCCTTCGGGAAGTTCG |
| Δ3_ext_R | CAGTACGGTCGATGGACCGG |
| Δ3_ext_R | GGAGATTGCCGGGGATACTC |
| Δ3_int_F | GCGACTTCCTGCTGGAAGAA |
| Δ3_int_R | GAATTCGAAGGCGTTACGCA |
| Δ6_ext_F | TGACCGCGGCGTTCGTCTTCTG |
| Δ6_ext_R | CAGCAGTTGCGCCTGGTTGTTG |
| Δ7_ext_F | GGGCTCAGCCACACGCGATAAC |
| Δ7_ext_R | CGACGATCTCGAACAGCGAACG |
| Δ14_1_ext_R | ATTCTCGCACGGAGGGATTC |
| Δ14_1_ext_R | TGAGCCAACCCCAGAAGAGT |
| Δ14_1_int_F | GAGCGTTGGCAGAGCGTGAT |
| Δ14_1_int_R | GCCCAAACCTTGTACTGCCC |
| Δ14_2_ext_F | TGGCCACCACCTGGCGCAGG |
| Δ14_2_ext_R | CCTGCGCCAGGTGGTGGCCA |
| Δ14_2_int_F | ACGGAAATCGAAGCGGATGC |
| Δ14_2_int_R | AGGCCGCGCTGATCTATGAA |
| Δ15_ext_F | ACGAAGCCATTGGCATCGAAC |
| Δ15_ext_R | CCCATTGAAACCGTACAGGC |
| Δ15_int_F | CGTCGACAATGAGGAACTGC |
| Δ15_int_R | CCGTATGTTCGGGTTGCTGA |
| Δ16_ext_F | CTACGCTTCGTTTCTGTGTC |
| Δ16_ext_R | TGCTGGAAAGCGAGTTCAGC |
| Δ17_ext_F | CATTACCTGAGCTTCCCAAGCT |
| Δ17_ext_R | GGCTGCGTCTCCGCCGAAATCC |
| Δ18_ext_F | CATTACCTGAGCTTCCCAAGCT |
| Δ18_ext_R | ACCCTGGCCCCTGAATCCACTC |
| rpsB1_F | GGCGGCATGCTCACCAACTA |
| rpsB1_R | ATCACGAACAGAGCGTCCGG |
| ORF1587_F | GTAGGGCCTTTTGCGACTCA |
| ORF1587_R | GATGTAAGATCAACGAGGGA |
| Ab09-F1 | GACGAAGCATCGTTGTACGC |
| Ab09-R1 | AACAACAATCGCAACTGGGC |
| Ab09ORF48_F1 | GCATGCGGCGGAAGAGTTCG |
| Ab09ORF48_R1 | AGGCTCAGGCTGAAGTGACC |
| Ab09ORF48_F2 | GCTGCACAGTGATAGTAGGC |
| Ab09ORF48_R2 | CCTACACTGGCCGTCTGTTC |
| ORF1587_BspHI_F | GCGCTCATGACGCACAACAAAATCTGTGTCAAAATCGCCTC |
| ORF1587_Hind3_R | GCGCAAGCTTCAGGAGCGATGCGGGGAATCCCCAGGGTAAC |
| rpsB _BspLU_F | GCGCACATGTCCCAAGTCAACATGCGCGATATGCTGAAGG |
| rpsB _Hind3_R | GCGCAAGCTTCAGCCTTCGGCGGATTCGGCCGGCGCTTCC |
|  |  |
